# Birth Outcome in Women with Previously Treated Breast Cancer—A Population-Based Cohort Study from Sweden

**DOI:** 10.1371/journal.pmed.0030336

**Published:** 2006-09-12

**Authors:** Kristina Dalberg, Johan Eriksson, Lars Holmberg

**Affiliations:** Department of Surgery and the Regional Oncologic Centre, Uppsala University Hospital, Uppsala, Sweden; Imperial College London, United Kingdom

## Abstract

**Background:**

Data on birth outcome and offspring health after the appearance of breast cancer are limited. The aim of this study was to assess the risk of adverse birth outcomes in women previously treated for invasive breast cancer compared with the general population of mothers.

**Methods and Findings:**

Of all 2,870,932 singleton births registered in the Swedish Medical Birth Registry during 1973–2002, 331 first births following breast cancer surgery—with a mean time to pregnancy of 37 mo (range 7–163)—were identified using linkage with the Swedish Cancer Registry.

Logistic regression analysis was used. The estimates were adjusted for maternal age, parity, and year of delivery. Odds ratios (ORs) and 95% confidence intervals (CIs) were used to estimate infant health and mortality, delivery complications, the risk of preterm birth, and the rates of instrumental delivery and cesarean section.

The large majority of births from women previously treated for breast cancer had no adverse events. However, births by women exposed to breast cancer were associated with an increased risk of delivery complications (OR 1.5, 95% CI 1.2–1.9), cesarean section (OR 1.3, 95% CI 1.0–1.7), very preterm birth (<32 wk) (OR 3.2, 95% CI 1.7–6.0), and low birth weight (<1500 g) (OR 2.9, 95% CI 1.4–5.8). A tendency towards an increased risk of malformations among the infants was seen especially in the later time period (1988–2002) (OR 2.1, 95% CI 1.2–3.7).

**Conclusions:**

It is reassuring that births overall were without adverse events, but our findings indicate that pregnancies in previously treated breast cancer patients should possibly be regarded as higher risk pregnancies, with consequences for their surveillance and management.

## Abstract 2

The large majority of births from women previously treated for breast cancer had no adverse events, but such pregnancies might benefit from increased surveillance and management.

## Editors' Summary

**Background.:**

More women of all ages are developing breast cancer than ever before. In the US, one woman in eight will now develop this disease during her lifetime. For most of these women, their breast cancer diagnosis will come late in life, but a fifth of breast cancers are diagnosed before the age of 50. These days, the long-term outlook for women with breast cancer is quite good; 80% of women who receive a diagnosis of breast cancer survive more than five years. These figures, together with a trend towards starting families later in life—since the late 1970s birth rates for women in their late 30s and 40s have more than doubled in the US, and in Sweden the average age for having a first baby is now 29 years—mean that many women who have had breast cancer want to have children. One estimate is that up to 7% of women who are fertile after treatment for breast cancer will later have children.

**Why Was This Study Done?:**

Pregnancy seems to have no adverse affects on women who have had breast cancer—there is no evidence that pregnancy can trigger a relapse. However, little is known about whether the chemotherapy and radiotherapy used to treat breast cancer have any long-lasting effects that might result in a poor birth outcome such as stillbirth, low birth weight, premature delivery, or abnormalities in the baby (congenital abnormalities). In this study, the researchers assessed the risk of adverse birth outcomes in women previously treated for breast cancer in Sweden.

**What Did the Researchers Do and Find?:**

Nearly three million singleton births that occurred between 1973 and 2002 are recorded in the Swedish Medical Birth Registry. The researchers linked this information with that in the Swedish Cancer Registry to identify 331 first births after treatment for invasive breast cancer (cancer that has spread from where it started to grow in the breast). The birth registry includes details on maternal age and health, child's birth weight, whether the delivery was preterm, and whether the child had any congenital abnormalities, so the researchers were able to compare birth outcomes in these 331 births with those in the general population. They discovered that most births after breast cancer treatment went smoothly. There was no increase in stillbirths, but there were slightly more delivery complications in the women who had had breast cancer than in the general population, and a slight increase in babies born prematurely or with low birth weight. Finally, a few more babies with congenital abnormalities were born to women after breast cancer treatment than to women in the general population.

**What Do These Findings Mean?:**

Overall, these results should reassure women who are thinking about having children after breast cancer about the health of their future offspring. However, they do suggest that these women may need careful monitoring during late pregnancy and delivery. This result was not predicted by the researchers who performed the study. Before starting the study, they thought that there would be no difference in birth outcomes between patients previously treated for breast cancer and the general population. Furthermore, a recently published similar study in Denmark found no increased risk of preterm birth, low birth weight, or congenital abnormalities after breast cancer. Differences between the two countries in the accuracy of their registries or in the use of chemotherapy and radiotherapy treatments may account for this difference in results. Additional studies are now needed in other populations to resolve this discrepancy and to provide more information about how breast cancer treatment might affect birth outcomes. For example, the current study did not provide any information about whether specific chemotherapy regimens or different types of breast cancer might put women at a higher risk of adverse birth outcomes, or whether the time between the cancer diagnosis and treatment and the pregnancy made a difference.

**Additional Information.:**

Please access these Web sites via the online version of this summary at http://dx.doi.org/10.1371/journal.pmed.0030336.

- MedlinePlus encyclopedia entry on breast cancer
- National Cancer Institute information for patients and physicians on breast cancer, including links to pages on breast cancer and pregnancy
- Cancer Research UK's information on breast cancer for patients, and statistics on breast cancer in the UK
• Wikipedia page on breast cancer (note: Wikipedia is a free online encyclopedia that anyone can edit)
- Royal College of Obstetricians and Gynaecologists guidelines for physicians on pregnancy and breast cancer

## Introduction

The average age of women giving birth for the first time has gradually increased during the last decades in Sweden. At present, the mean age at first birth is 29 y. The incidence of breast cancer is increasing in women of all ages.

Between 20% and 25% of breast cancer diagnoses are in women under 50 y [1,2]. Some of these women face diagnosis and treatment of breast cancer before they have started or completed childbearing and need to be appropriately counseled.

Knowledge about how breast cancer and its treatment affect birth outcome and offspring health is limited. In a recently published Danish cohort study [3] there was no excess risk of adverse birth outcome for 216 newborns of women with breast cancer before pregnancy compared with women without breast cancer.

The aim of the present study was to assess delivery risks and offspring health for births by previously treated breast cancer patients compared with the general population. When the study was initiated we hypothesized, based on the results of two clinical reports [4,5] and our clinical experience, that no difference would be detected.

## Methods

The study is based on 2,870,932 singleton births, registered in the Swedish Medical Birth Registry during 1973–2002. Linking this register with the Swedish Cancer Registry, 414 births were identified of women with previously treated invasive breast cancer. Invasive breast cancer was defined according to World Health Organization's histological typing of breast tumors. In total, 331 of the 414 births were the first births subsequent to breast cancer diagnosis, with a mean time between breast cancer surgery and pregnancy of 37 mo (range 7–163). Second and third births subsequent to breast cancer diagnosis were excluded from the analysis in order to analyze one birth linked to each woman exposed to breast cancer who subsequently gave birth. In women without breast cancer, all singleton births were included.

The Swedish Medical Birth Registry of the National Board of Health Welfare has data from over 99% of all births in Sweden since 1973 [6]. Starting with the first antenatal visit, antenatal, obstetric, and pediatric information are recorded in a standardized manner.

Maternal age was defined as age in completed years at the time of delivery. Parity was defined as number of previous births, including stillbirths. Information about maternal smoking was collected at the first antenatal visit. Women were classified in this study as nonsmokers or daily smokers (one or more cigarettes per day). The woman's country of birth was also recorded, and whether the woman was living with the child's father.

During the period of 1973–2002, three different editions of the International Classification of Diseases were used (ICD-8: 1955–1986; ICD-9: 1987–1990; and ICD 10: 1991–2002).

The variable hypertension was defined as essential hypertension (diagnosed before pregnancy and registered as a separate variable at the first antenatal visit), gestational hypertension, and/or preeclampsia/eclampsia using the following one or more diagnostic codes: ICD-8: 401 and 637; ICD-9: 642; and ICD-10: O10–O16.

Diabetes was defined as insulin-dependent diabetes present before pregnancy, non-insulin-dependent diabetes present before pregnancy, or gestational diabetes (ICD-8: 250; ICD-9: 250 and 648; and ICD-10: O24).

Complications during pregnancy and delivery were classified by a physician when the woman was discharged. For an outcome of pregnancy bleeding we used ICD-8: 632; ICD-9: 640–641; and ICD-10: O20 and O46; and for deliveries complicated by placenta previa, ante- or postpartum hemorrhage, retained placenta, abnormal bone pelvis, fetopelvic disproportion, malpresentation of fetus, prolonged labor, rupture of uterus, laceration of perineum, or other complications we used ICD-8: 651–662; ICD-9: 652–669; and ICD10: O45–O75. To register birth injury we used ICD-8: 772; ICD-9: 767; and ICD-10: P10–P15; and to register both major and minor malformations we used ICD-8: 740–759; ICD-9: 740–759; and ICD-10: Q0–Q99. All diagnostic codes were registered as none or one or more.

Estimated gestational age was based on the first day of the last menstruation and/or on ultrasound examination performed routinely at no later than 18 completed weeks of gestation. During the two last decades, anomaly screening ultrasound has been uniformly performed in Sweden.

The definition of a live birth was a newborn with a gestational age of 22 wk or more that showed any evidence of life, such as beating of the heart, pulsation of the umbilical cord, or definite movement of voluntary muscles. Stillbirth was defined as late fetal death occurring at 28 or more completed weeks of gestation. Preterm delivery was classified as very preterm (≤32 wk) or preterm (33 to 36 wk).

Infants defined as small for gestational age were those with birth weights more than two standard deviations below the mean birth weight for gestational age according to a Swedish reference curve.

Apgar score at 5 min was categorized as (1) below seven or (2) seven or greater because a previous population-based study in Sweden showed that a 5-min Apgar score under seven in term infants was associated with an increased risk of neonatal morbidity and neurologic impairment [7].

The study was approved by the Ethical Committee for Human Studies of Uppsala University.

The present study uses a cohort design and logistic regression analysis to estimate odds ratios (ORs) for birth outcome. The exposure of interest was breast cancer prior to birth, and the events adverse birth outcomes as defined above. We considered age of mother, time period of delivery, country of mother's birth, history of infertility, family situation, smoking habits, hypertension, and diabetes to be possible confounders. After studying the distribution of these variables among women exposed to breast cancer those not, we kept age of mother, parity, and year of delivery as variables of interest—both as possible confounders and theoretically also as modifiers of the effect. We started the analysis by stratifying on age of mother, parity, and year of delivery, in periods of 5-y intervals. All crude estimates regarding birth outcome and infant characteristics were adjusted for age of mother (continuous), parity, and year of delivery. When there was a binary response variable, the logistic procedure with canonical (i.e., logit) link in SAS version 8.2 (SAS Institute, http://www.sas.com) was used to calculate the OR and its 95% confidence interval (CI). When there was a multinomial response variable, the generalized (glogit) link function was used [8]. No adjustments were made for multiple tests.

## Results

The information on parity and maternal age was complete for all individuals (Table 1). Women formerly exposed to breast cancer (the BC group) were older at delivery, with a mean age of 34 y, than the general population of mothers (mean age 27 y), and more often had a history of infertility lasting more than 1 y (OR 2.53, 95% CI 1.6–3.9). Parity and frequencies of hypertensive disease, smoking, living with the father, and diabetes were comparable between the BC group and women not exposed to breast cancer.

**Table 1:**
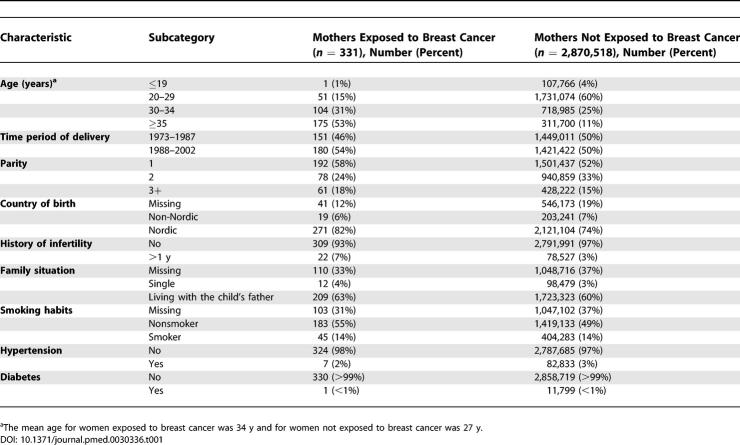
Characteristics of Women Delivering the First Infant Subsequent to Breast Cancer Compared with the General Population of Women Delivering Infants

The OR for delivery complications was increased among women in the BC group (Table 2). Delivery complications were registered as none or one or more. Thirty-one percent of infants of women in the BC group were delivered by instrument or by cesarean section, compared to 17% in women not exposed to breast cancer.

**Table 2:**
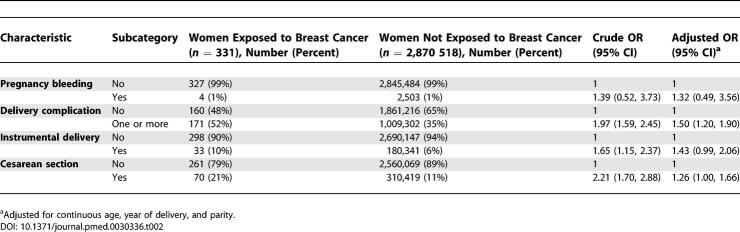
Crude and Adjusted ORs for Birth Outcome in Women Previously Treated for Breast Cancer Compared with the General Population

The characteristics of the infants born to the 331 women in the BC group compared with women without breast cancer are shown in Table 3. Eleven percent of the infants of women in the BC group were delivered preterm (before week 37), compared with 5% of infants born to women without a previous breast cancer.

**Table 3:**
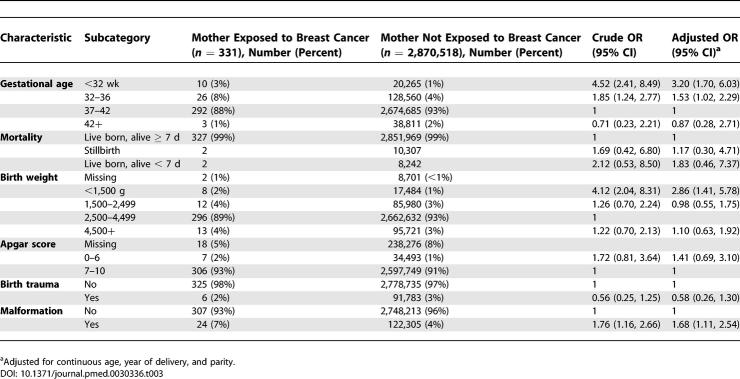
Characteristics of Infants Born to Women Previously Treated for Breast Cancer Compared with the General Population of Infants Born

We found no increased risk of reduced birth weight for gestational age in infants of mothers in the BC group: the OR for infants being small for their gestational age was 1.2 (95% CI 0.90–1.39) compared to the general population of infants.

The OR for delivering an infant with an Apgar score below seven at 5 min was not increased among women in the BC group.

Malformation, major or minor, was registered in 7.5% of the infants of women in the BC group compared to 4.3% among mothers without a previous breast cancer (Table 3). A tendency towards an increased risk of malformations in the infants of mothers who have had breast cancer was seen especially in the later time period of births (1988–2002)—OR 2.1 (95% CI 1.2–3.7)—in contrast to the first time period (1973–1987), with an OR of 1.3 (95% CI 0.7–2.5).

In infants of women in the BC group, nine malformations were registered between 1973 and 1987 and 15 between 1988 and 2002. The diagnoses in these 24 offspring were as follows: ten cardiac defects (including three children with patent ductus arteriosus and four with septal defects), three kidney/ureteragenesis defects, two undescended testes in full-term infants, two unspecified limb malformations, two ear malformations, two skin malformations, one chromosome anomaly (trisomy 21), one congenital hydrocephaly, and one orofacial cleft. Only one malformation diagnosis for each infant was found.

## Discussion

In the present study, with 331 births subsequent to breast cancer, the large majority of infants were born alive, full term, with Apgar scores at 5 min of seven or more, and with no malformation. Regarding stillbirth and infant death—arguably the most serious adverse outcomes—the result of the current study is reassuring. Only four deaths in 331 births of mothers in the BC group were identified. Still, we found a slightly increased risk of adverse pregnancy outcomes, such as an increased risk for delivery complications, cesarean section, preterm birth, and low birth weight. These associations remained almost unchanged after adjustment for possible maternal confounders such as age and parity.

The strengths of the study are that it is population-based, covers a large number of pregnancies over a long time period, and has no losses to follow-up, and that the registries have near complete information on all key variables. The Swedish Cancer Registry has over 95% sensitivity in registration of all breast cancers in the country.

A drawback of the study approach is that we have no information for individuals on the type of treatment for breast cancer they received, or on the timing and course of the disease with respect to the pregnancy. Thus, we cannot define subgroups with higher or lower risks within those exposed to breast cancer. We also had no data on and therefore could not control for maternal use of antidepressants, reported to be associated with preterm birth [9]. There is no information available on whether pregnant women with a previous breast cancer use antidepressants more often than other pregnant women. Another limitation of the study is the possibility of misclassification of the outcome variables: during the study period three different ICD classifications of diagnoses were used, which could lead to registration artifacts.

There have been studies of the accuracy of the data on pregnancy and malformations in the Swedish Medical Birth Registry [10–12]. A questionnaire on pregnancy outcome was distributed to 782 women in Sweden [10]. Only 50% of self-reported less severe malformations were found in the central registry, but the agreement between the questionnaire data and the registry was good for serious malformations. Regarding birth weight, there was agreement in 72% of cases.

However, most likely any pregnancy and birth misclassifications are non-differential with regard to a previous breast cancer diagnosis on the part of the mother, which, if anything, will lead to an underestimation of the ORs rather than spurious positive findings. The increased use of cesarean section in women with breast cancer may, however, very well be a consequence of increased fetal monitoring.

As this is a birth registry study, major malformations that led to pregnancy termination would not be counted in either group. However, it is unlikely that the difference in severe malformations reported here is a consequence of surveillance or diagnostic bias in this study, since a serious malformation would be regarded as a major medical event in all women and not easily overlooked.

Healthy offspring have been reported delivered from small series of breast cancer patients [4,5], and a recent large Danish cohort study [3] also has shown reassuring results. No increased risk of preterm birth, low birth weight, stillbirth, or congenital abnormalities was seen in 216 infants born in 1943–2002 of women with breast cancer before pregnancy. There may be several reasons why the results differ between the Danish and the present Swedish cohort. There may be different degrees and patterns of misclassification of the outcome variables between the registries. There may be differences in the usage of adjuvant radiotherapy or systemic treatments after breast cancer. The time trend in risk for malformations seen in our study supports the notion that chemotherapy may be a driving force behind the risks, as use of chemotherapy in younger patients has increased sharply since 1988.

As the incidence of breast cancer in women less than 50 y of age is increasing, data about the impact of adjuvant therapy on reproductive potential and offspring outcome are needed. Cytotoxic agents are preferentially toxic to rapidly dividing cells and demonstrate teratogenic effects that can cause malformations when administered in the first trimester of pregnancy [13]. During the second and third trimester, fetal growth and functional development, especially that of the brain, may be affected [13]. No increase in birth defects has been demonstrated in children whose parents were exposed to chemotherapy earlier in life [5,14–16].

Little is known about the late effects of chemotherapy on offspring. Mulvihill et al. [17] reported on 58 pregnancies occurring with a mean time of 27 mo after chemotherapy for various malignancies. During the first year after chemotherapy, an increase in low birth weight—mainly due to premature birth—stillbirth, and premature termination of pregnancy was seen, but no excess of congenital anomalies.

Having a malignancy and subsequent pregnancy raises questions about the mother and her disease, on the one hand, and the management of the fetus, on the other. Breast cancer survivors who want to become pregnant need information about potential risks. Breast cancer patients' prognosis does not seem to be worsened by pregnancy [18–20]. The knowledge of potential risks for delivery offspring health compared to that of the general population has so far been scanty. Furthermore, there are few studies in which the characteristics of pregnant breast cancer patients are compared with those of the general population.

We hypothesized wrongly that no increased risk of adverse birth outcomes would be detected. While it should be pointed out that the large majority of births had no complications, one may consider that based on our observations, pregnancies in previously treated breast cancer patients should be regarded as higher risk pregnancies, with consequences for their surveillance and management.

## Abbreviations

CI: confidence interval
OR: odds ratio
